# Exposure to Parasitic Protists and Helminths Changes the Intestinal Community Structure of Bacterial Communities in a Cohort of Mother-Child Binomials from a Semirural Setting in Mexico

**DOI:** 10.1128/mSphere.00083-21

**Published:** 2021-08-18

**Authors:** Oswaldo Partida-Rodriguez, Miriam Nieves-Ramirez, Isabelle Laforest-Lapointe, Eric M. Brown, Laura Parfrey, Alicia Valadez-Salazar, Lisa Thorson, Patricia Morán, Enrique Gonzalez, Edgar Rascon, Ulises Magaña, Eric Hernandez, Liliana Rojas-Velázquez, Javier Torres, Marie Claire Arrieta, Cecilia Ximenez, B. Brett Finlay

**Affiliations:** a Laboratorio de Inmunología del Unidad de Medicina Experimental, UNAM, Mexico City, Mexico; b Michael Smith Laboratories, Department of Microbiology and Immunology, University of British Columbia, Vancouver, British Columbia, Canada; c Department of Physiology and Pharmacology, University of Calgary, Calgary, Alberta, Canada; d Department of Pediatrics, University of Calgary, Calgary, Alberta, Canada; e Département de Biologie, Université de Sherbrooke, Sherbrooke, Québec, Canada; f Department of Zoology, University of British Columbia, Vancouver, British Columbia, Canada; g Department of Botany, University of British Columbia, Vancouver, British Columbia, Canada; h Unidad de Investigación en Enfermedades Infecciosas, UMAE Pediatria, IMSS, Mexico City, Mexico; i Department of Biochemistry and Molecular Biology, University of British Columbia, Vancouver, British Columbia, Canada; j Department of Microbiology and Immunology, University of British Columbia, Vancouver, British Columbia, Canada; National Institute of Advanced Industrial Science and Technology

**Keywords:** parasites, eukaryotes, protists, helminths, microbiota, bacteria, 16S sequencing, 18S sequencing, children, Mexico

## Abstract

An estimated 3.5 billion people are colonized by intestinal parasites worldwide. Intestinal parasitic eukaryotes interact not only with the host but also with the intestinal microbiota. In this work, we studied the relationship between the presence of multiple enteric parasites and the community structures of gut bacteria and eukaryotes in an asymptomatic mother-child cohort from a semirural community in Mexico. Fecal samples were collected from 46 mothers and their respective children, with ages ranging from 2 to 20 months. Mothers and infants were found to be multiparasitized by Blastocystis hominis, Entamoeba dispar, Endolimax nana, Chilomastix mesnili, Iodamoeba butshlii, Entamoeba coli, Hymenolepis nana, and Ascaris lumbricoides. Sequencing of bacterial 16S rRNA and eukaryotic 18S rRNA genes showed a significant effect of parasite exposure on bacterial beta-diversity, which explained between 5.2% and 15.0% of the variation of the bacterial community structure in the cohort. Additionally, exposure to parasites was associated with significant changes in the relative abundances of multiple bacterial taxa, characterized by an increase in *Clostridiales* and decreases in *Actinobacteria* and *Bacteroidales*. Parasite exposure was not associated with changes in intestinal eukaryote relative abundances. However, we found several significant positive correlations between intestinal bacteria and eukaryotes, including *Oscillospira* with *Entamoeba coli* and Prevotella stercorea with Entamoeba hartmanni, as well as the co-occurrence of the fungus *Candida* with *Bacteroides* and *Actinomyces*, *Bifidobacterium*, and Prevotella copri and the fungus *Pichia* with *Oscillospira*. The parasitic exposure-associated changes in the bacterial community structure suggest effects on microbial metabolic routes, host nutrient uptake abilities, and intestinal immunity regulation in host-parasite interactions.

**IMPORTANCE** The impact of intestinal eukaryotes on the prokaryotic microbiome composition of asymptomatic carriers has not been extensively explored, especially in infants and mothers with multiple parasitic infections. In this work, we studied the relationship between protist and helminth parasite colonization and the intestinal microbiota structure in an asymptomatic population of mother-child binomials from a semirural community in Mexico. We found that the presence of parasitic eukaryotes correlated with changes in the bacterial gut community structure in the intestinal microbiota in an age-dependent way. Parasitic infection was associated with an increase in the relative abundance of the class *Clostridia* and decreases of *Actinobacteria* and *Bacteroidia*. Parasitic infection was not associated with changes in the eukaryote community structure. However, we observed strong positive correlations between bacterial and other eukaryote taxa, identifying novel relationships between prokaryotes and fungi reflecting interkingdom interactions within the human intestine.

## INTRODUCTION

Bacteria, viruses, archaea, fungi, and protists inhabiting the mucosal surfaces of the human body have coevolved with the human intestine for millions of years and have broad effects on the physiology of the host. The diverse intestinal bacterial community shares its habitat with a dynamic community of eukaryotes, many of which are well-known parasites. Their interaction can affect the success of parasite colonization, influencing its outcome along the entire parasitism-mutualism spectrum (1). Furthermore, these microbial communities can affect host processes, including metabolism and the normal development and function of the mucosal and systemic immune systems (2–10).

Even though parasites colonize millions of people around the world, the potential damage caused by parasitic colonization is often controlled either by the host or by both the parasite and host, leading to asymptomatic colonization. The influence of intestinal parasites on resident bacterial and eukaryotic community structures has not been fully addressed; some of the previous works have been performed in experimental models of disease (10), but studies in the human host are scarce. Thus, the study of how parasites influence the intestinal microbiota in asymptomatic individuals is an important and relevant topic, especially given the large effect that the microbiome has on the host.

Little is known about the eukaryotic microbiome, or “eukaryome,” and virtually nothing is known about its community structure, particularly in the case of protist colonizations. Morton et al. (5) found a strong association of the presence of *Entamoeba* with a higher frequency of *Firmicutes* and a lower frequency of *Bacteroidetes* in Entamoeba histolytica-positive samples. Nieves-Ramírez et al. (11), working in the same semirural Mexican population as the one of this study, found that asymptomatic colonization in adults with the protist *Blastocystis* was strongly associated with increases in alpha- and beta-diversities of bacteria and with more discrete changes in the microbial eukaryome.

It has been widely documented that the initial development of the intestinal microbiota in infants has a profound effect on adult intestinal health and disease (12–16). The first 1,000 days of life play an important role in determining the phylogenetic structure of the adult human gut microbiota (16) and factors such as birth mode, breastfeeding, geographical location, household siblings, and pets are important in intestinal microbiome development, especially during the first year of life (17, 18). Early postnatal exposures to parasitic infections could also affect the development of the gut microbiota structure. Given the lack of knowledge on the role of eukaryotes in the establishment of the early-life microbiota, we aimed to study this in a population of 46 mother-child (M-C) asymptomatic binomials from a semirural Mexican population with high levels of intestinal parasite exposure. A comprehensive microbiome assessment was performed using 16S rRNA and 18S rRNA Illumina sequencing analyses for the characterization of bacteria and eukaryotes in the fecal microbiome. The results revealed interesting associations between parasite colonization and distinct microbiome patterns in the intestines of children under 2 years of age.

## RESULTS

### Characteristics of the individuals studied.

A cohort of 46 mother-child (M-C) binomials was studied. For the study, we selected 11 parasite-positive weaned infants and their parasite-positive mothers, 13 parasite-negative weaned infants and their parasite-negative mothers, 12 parasite-exposed unweaned infants (parasite-negative children) and their parasite-positive mothers, and 10 parasite-unexposed unweaned infants (negative children) and their parasite-negative mothers (Table 1).

**TABLE 1:** Demographic data for the children in this study

Parameter	Value for group					
	Unweaned			Weaned		
	Parasite exposed (*n* = 12)	Parasite unexposed (*n* = 10)	*P* value^*a*^	Parasite positive (*n* = 11)	Parasite negative (*n* = 13)	*P* value^*a*^
Mean age (mo)	3.0	3.1	0.6951	19.9	17.1	0.0189
Mean age at weaning (mo)				11.5	10.5	0.4010
No. of females/no. of males	3/9	5/5	0.2248	9/2	3/10	0.0041
No. of children delivered vaginally/no. delivered by C-section	12/12	10/10		5/6	7/6	0.6820
Mean age of mother (yrs)	25.5	29.5	0.0839	25.1	29.5	0.1225

a Statistical analysis was performed using chi-square and Fisher’s exact tests for categorical data and a *t* test for numerical data.

The age of the children ranged from 2 to 20 months (average of 11.04 months). The parasite-positive weaned children were significantly older (*P* = 0.0189) and were composed of more females (*P* = 0.0041) than the parasite-negative weaned children. Children were breastfed for an average of 10.9 months (6 to 12 months). Twelve children were delivered by C-section, and 34 were delivered vaginally. All individuals were within a healthy weight range and did not show any stunting or wasting. The mothers’ average age was 27.4 years, with a range of 18 to 47 years. None of the participants reported antibiotic or other drug use, gastrointestinal symptoms (according to the Rome III questionnaire), or inflammatory signs and symptoms (according to medical examination) in the 6 months prior to sampling.

### Parasites found in mother-child binomials.

**FIG 1:**
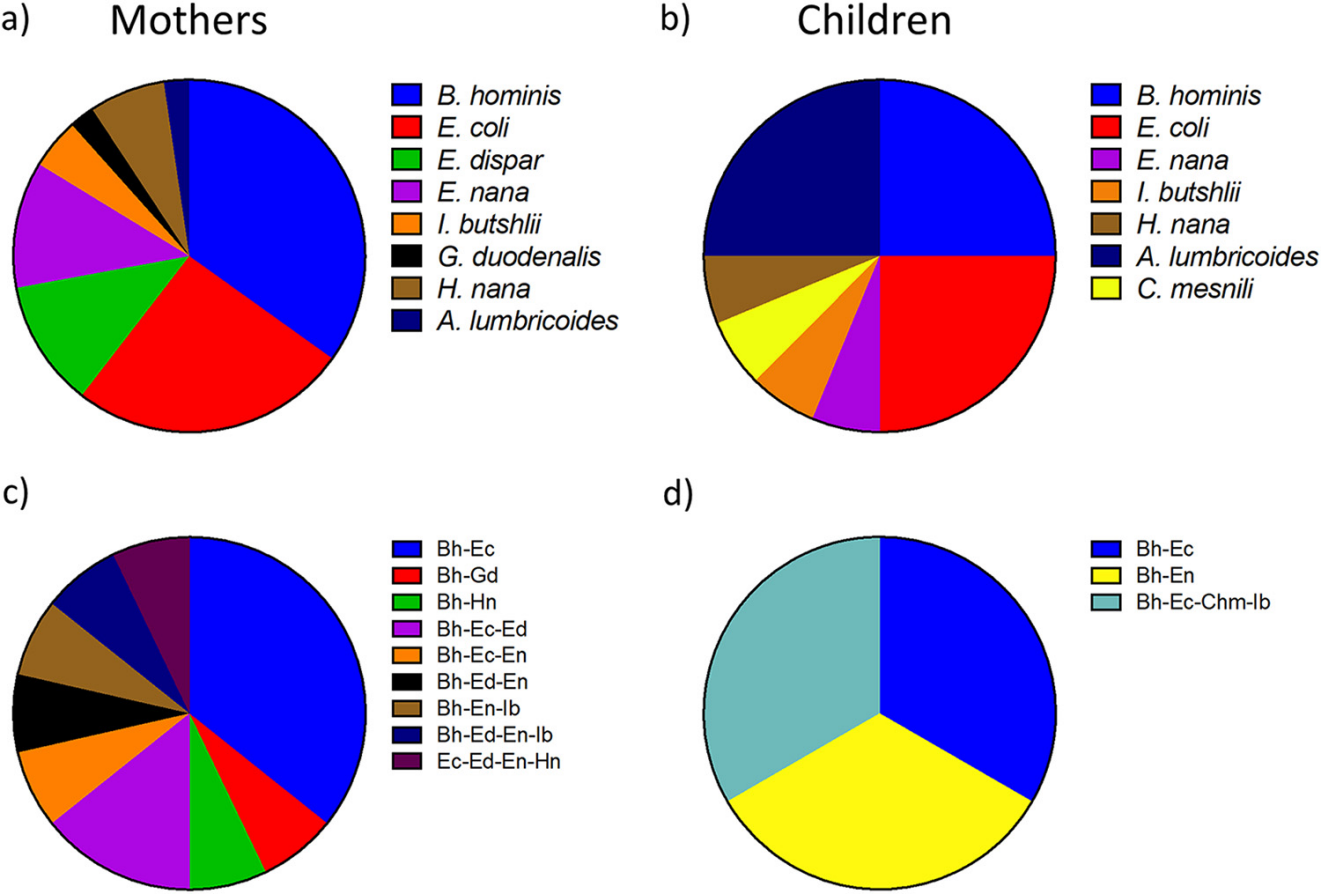
Proportions of parasites found in fecal samples from mother (a and c)-weaned child (b and d) binomials. (a) The intestinal parasites found in the mothers comprised *Blastocystis hominis* (Bh) (15 [32.6%]), *Entamoeba coli* (Ec) (11 [23.9%]), Entamoeba dispar (Ed) (5 [10.8%]), *Endolimax nana* (En) (5 [10.8%]), *Iodamoeba butshlii* (Ib) (2 [4.3%]), Giardia duodenalis (Gd) (1 [2.2%]), *Chilomastix mesnili* (Chm) (0 [0.0%]), *Hymenolepis nana* (Hn) (3 [6.5%]), and *Ascaris lumbricoides* (Al) (1 [2.2%]). (b) In children, the parasite frequencies were determined for *Blastocystis hominis* (4 [8.7%]), *Entamoeba coli* (4 [8.7%]), Entamoeba dispar (0 [0.0%]), *Endolimax nana* (1 [2.2%]), *Iodamoeba butshlii* (1 [2.2%]), Giardia duodenalis (0 [0.0%]), *Chilomastix mesnili* (1 [2.2%]), *Hymenolepis nana* (1 [2.2%]), and *Ascaris lumbricoides* (4 [8.7%]). (c) Cocolonizations by two or more parasites found in the mothers included *Blastocystis hominis*-*Entamoeba coli* (5 [10.8%]), *Blastocystis hominis*-Giardia duodenalis (1 [2.2%]), *Blastocystis hominis*-*Hymenolepis nana* (1 [2.2%]), *Blastocystis hominis*-*Entamoeba coli*-Entamoeba dispar (2 [4.3%]), *Blastocystis hominis*-*Entamoeba coli*-*Endolimax nana* (1 [2.2%]), *Blastocystis hominis*-Entamoeba dispar-*Endolimax nana* (1 [2.2%]), *Blastocystis hominis*-*Endolimax nana*-*Iodamoeba butshlii* (1 [2.2%]), *Blastocystis hominis*-Entamoeba dispar-*Endolimax nana*-*Iodamoeba butshlii* (1 [2.2%]), and *Entamoeba coli*-Entamoeba dispar-*Endolimax nana*-*Hymenolepis nana* (1 [2.2%]). (d) Cocolonizations found in children were *Blastocystis hominis*-*Entamoeba coli* (1 [2.2%]), *Blastocystis hominis*-*Endolimax nana* (1 [2.2%]), and *Blastocystis hominis*-*Entamoeba coli*-*Chilomastix mesnili*-*Iodamoeba butshlii* (1 [2.2%]).

Intestinal parasites found by either microscopy or quantitative PCR (qPCR) in feces of mothers and children included Blastocystis hominis, Entamoeba coli, Entamoeba dispar, Endolimax nana, Iodamoeba butshlii, Giardia duodenalis, Chilomastix mesnili, Hymenolepis nana, and Ascaris lumbricoides (Fig. 1). E. dispar was distinguished from E. histolytica in the taxonomy assignment from 18S sequencing. The parasite most frequently found was B. hominis, present in 32.6% of positive mothers and 8.7% of positive children, followed by E. coli (23.9% and 8.7%, respectively). In the mothers, it was also common to find *E. dispar* (10.8%), E. nana (10.8%), and H. nana (6.5%). In contrast to mothers, A. lumbricoides was among the most frequent parasites in infants (8.7%). We also found several cases of cocolonizations by two or more intestinal parasites, particularly in the mothers, with *B. hominis-*E. coli being the most frequently found (10.8% in mothers and 2.2% in infants). Of note, none of the parasite-positive individuals (either mothers or infants) presented gastrointestinal symptoms.

### Bacterial and eukaryotic diversity in individuals colonized by parasites.

**(i) Binomial identity.** We determined the fecal bacterial and eukaryotic compositions of the 46 binomials. The parasites found by microscopy or qPCR that were also detected by 18S rRNA gene sequencing were *B. hominis*, E. coli, *E. dispar*, *E. nana*, *Iodamoeba*, *Ascaris*, and *H. nana*.

In order to evaluate if the mother’s intestinal microbiota composition could be a determinant of the intestinal microbiota in children, we tested for differences in Bray-Curtis distances between binomial pairs (intrabinomial) versus all other individuals (interbinomial) with a nonparametric Kruskal-Wallis test. Intrabinomial dissimilarities were not significantly different from interbinomial dissimilarities for both bacterial and eukaryote gut communities (see Fig. S1 in the supplemental material).

10.1128/mSphere.00083-21.1 FIG S1 Principal-component analysis (PCoA) ordination of the variation in the beta-diversity of human gut bacterial (left) and eukaryote (right) communities in weaned infant (1 year old)-mother binomials (a and b) and unweaned infant (3 months old)-mother binomials (c and d) on Bray-Curtis dissimilarities. Colors represent binomial identity, while shapes represent class (squares for mothers and circles for infants). PERMANOVAs indicate no significant effect of binomial identity. Dark colors indicate no colonization, while light colors indicate colonization. Download FIG S1, TIF file, 0.6 MB. Copyright © 2021 Partida-Rodriguez et al. 2021 Partida-Rodriguez et al. https://creativecommons.org/licenses/by/4.0/ This content is distributed under the terms of the Creative Commons Attribution 4.0 International license.

**(ii) Effects on infants less than 5 months old.** Because of the known differences in microbiome structure and diversity across the first year of life, as well as due to breastfeeding as a major factor influencing microbiome development over this period (17, 18), we evaluated the effect of parasites in unweaned infants under 1 year of age separately from the rest of the individuals over 1 year of age, including weaned infants. The infants under 1 year of age were all under 5 months of age, and none of them were infected by parasites, but we considered them parasite exposed when their mothers tested positive for parasites.

**FIG 2:**
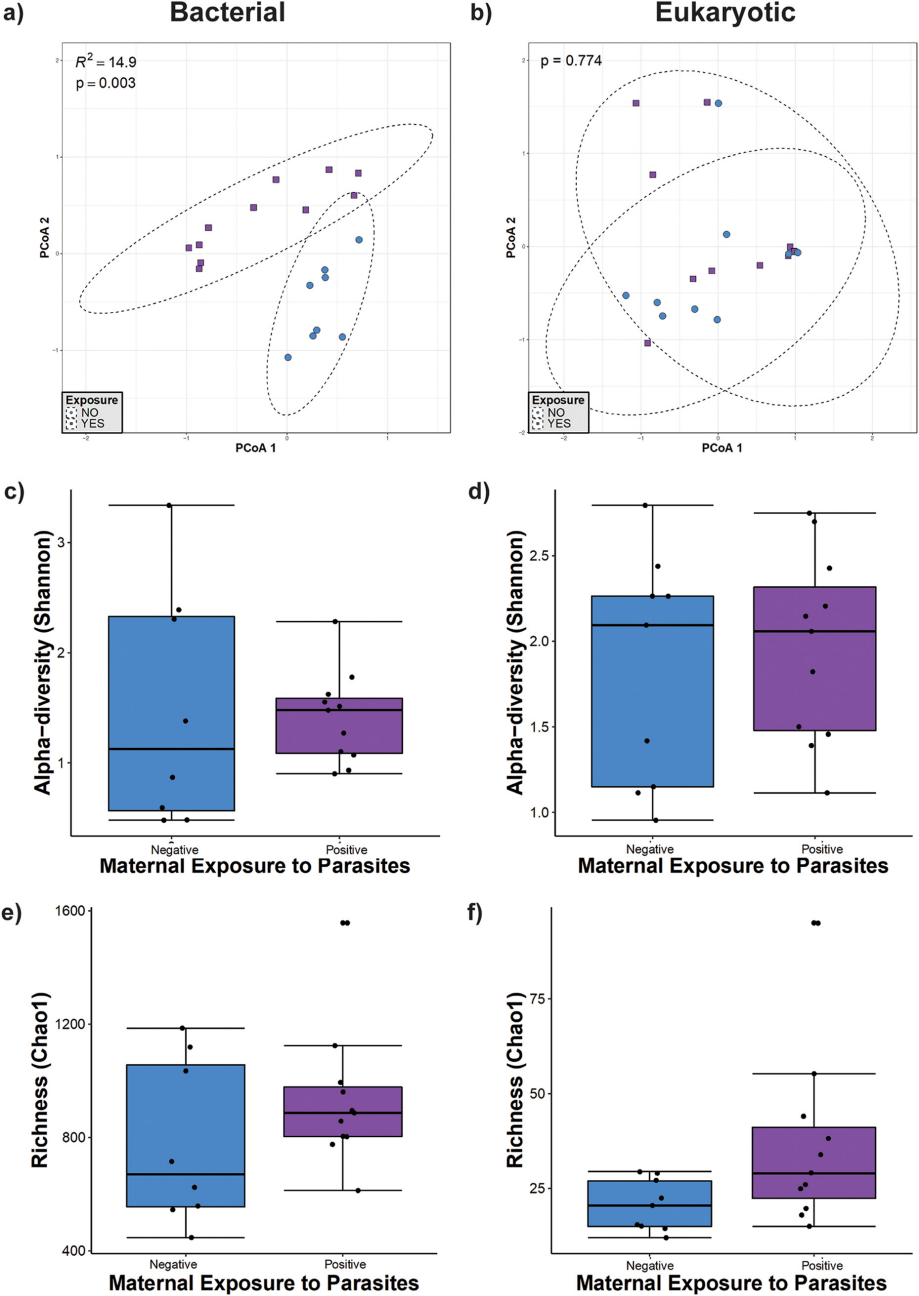
Microbial diversity in unweaned infants (younger than 5 months of age). (a and b) Principal-component analysis (PCoA) ordination of variation in beta-diversities of human gut bacterial (a) and eukaryote (b) communities based on Bray-Curtis dissimilarities. Color and shape represent maternal exposure to parasites (blue circles represent negative exposure, and purple squares represent positive exposure). PERMANOVAs indicate that maternal exposure to parasites explains 15% (*P* = 0.003) of the variation in the infant bacterial community structure but is not a significant (*P* = 0.774) driver of the eukaryote community structure. (c and d) Shannon diversity of gut bacterial (c) and eukaryote (d) community structures. (e and f) Estimated richness of gut bacterial (e) and eukaryote (f) community structures. No significant differences were detected by Mann-Whitney tests for alpha-diversity comparisons between the parasite-positive and -negative groups.

We identified the relationships between bacterial community structure and parasite exposure by conducting permutational multivariate analysis of variance (PERMANOVA) on the community matrix. Based on Bray-Curtis dissimilarities (Fig. 2), we found that the bacterial community structure was significantly related to parasite exposure in infants under 1 year of age (*P* = 0.003 by PERMANOVA). According to PERMANOVA, parasite exposure explained 15% of the variation in bacterial beta-diversity (Fig. 2a); however, we did not find an effect of parasite exposure on the eukaryote community structure (Fig. 2b). We also calculated the Shannon diversity index and Chao1 richness of bacterial and eukaryotic communities in this group of infants, and we did not find any statistically significant differences between the exposed and nonexposed groups (Fig. 2c to f).

**FIG 3:**
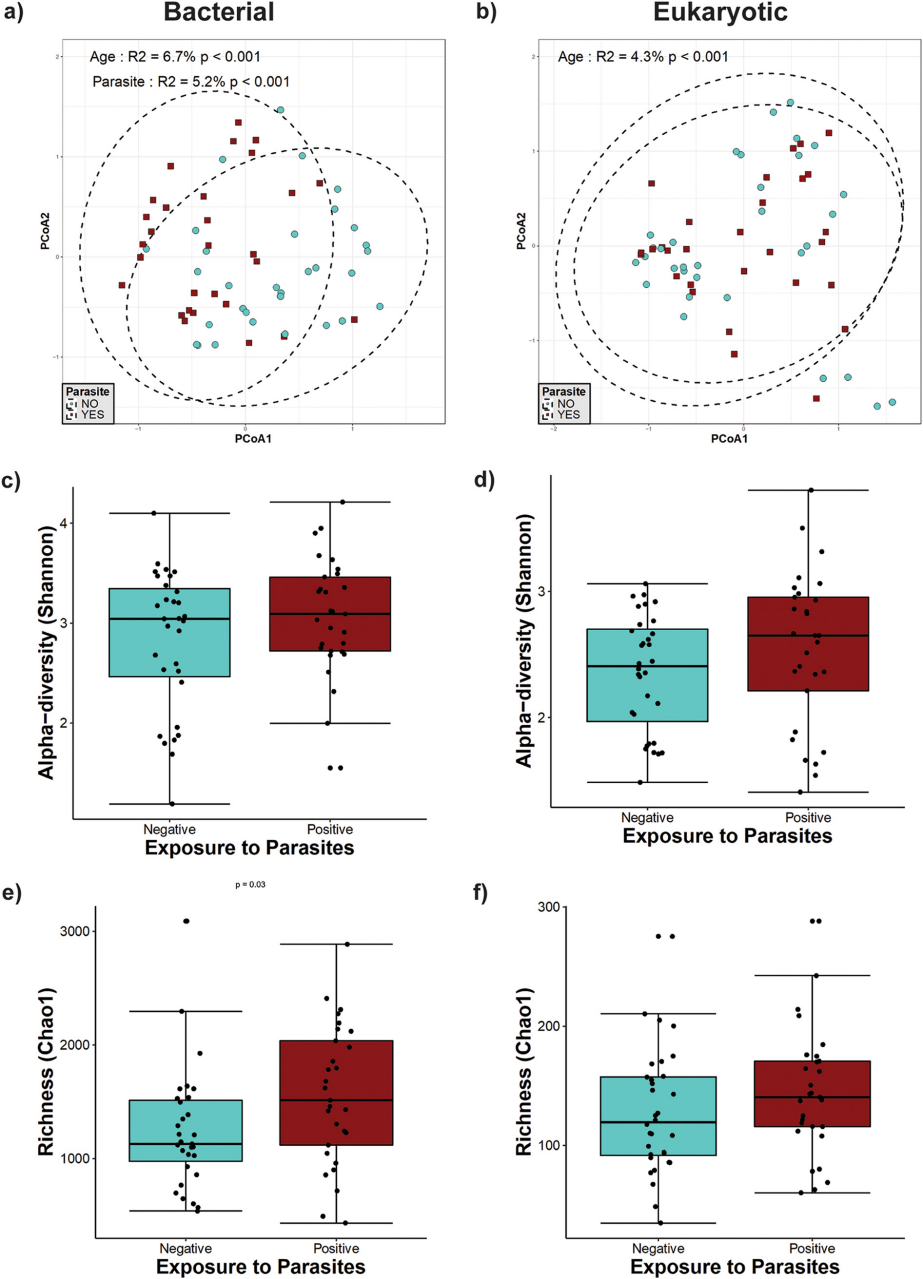
Microbial diversity in individuals older than 1 year of age. (a and b) Principal-component analysis (PCoA) ordination of the variation in the beta-diversity of human gut bacterial (a) and eukaryote (b) communities based on Bray-Curtis dissimilarities. Color and shape represent maternal exposure to parasites (turquoise circles for negative and dark-red squares for positive exposure). PERMANOVAs indicate that maternal exposure to parasites and age explain 5.2% and 6.7% (*P* < 0.001) of the variation in the bacterial community structure, respectively, while age explains 4.3% (*P* < 0.001) of the variation in the eukaryote community structure. Ellipses represent the confidence intervals at 95%. (c and d) Shannon diversity of gut bacterial (c) and eukaryote (d) community structures. No significant differences were detected by Mann-Whitney tests for Shannon diversity between the parasite-positive and -negative groups. (e and f) Estimated richness of gut bacterial (e) and eukaryote (f) community structures. A significant difference was detected for bacterial community richness by Mann-Whitney tests for comparisons between two groups.

**(iii) Individuals older than 1 year of age.** In individuals older than 1 year of age (including mothers and weaned children), we found a significant effect of parasite presence on bacterial beta-diversity (Fig. 3a), which explained 5.2% of the variation present in this group. The presence of intestinal parasites was also significantly associated with changes in richness, with an increase in the Chao1 richness for bacterial diversity (*P* = 0.04) but not eukaryotic diversity (Fig. 3e and f). However, there were no statistically significant differences in bacterial and eukaryote Shannon diversities between parasite-positive and -negative individuals in this age group (Fig. 3c and d), suggesting that changes in beta-diversity are mainly due to a modification of the bacterial taxa present in the gut microbiota. We also observed a significant effect of age on both bacterial (*P* < 0.001) (Fig. 3a) and eukaryote (*P* < 0.001) (Fig. 3b) beta-diversities, explaining the variation found in 6.7% and 4.3%, respectively.

**FIG 4:**
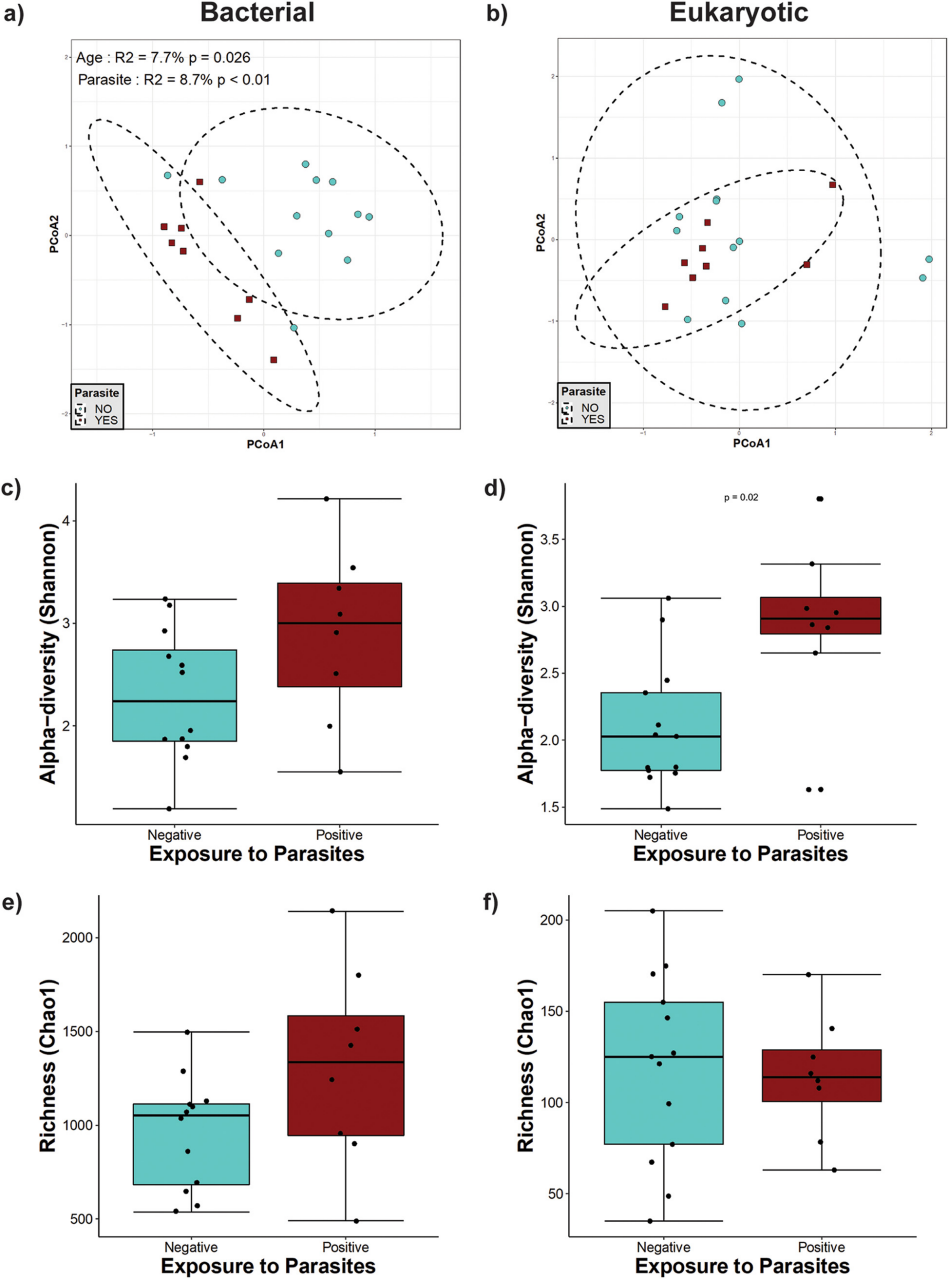
Microbial diversity in weaned infants between 1 year and 2 years of age. (a and b) Principal-component analysis (PCoA) ordination of the variation in the beta-diversity of human gut bacterial (a) and eukaryote (b) communities based on Bray-Curtis dissimilarities. Color and shape represent maternal exposure to parasites (turquoise circles for negative and dark-red squares for positive exposure). PERMANOVAs indicate that exposure to parasites and age explain 8.7% (*P* < 0.01) and 7.7% (*P* = 0.026) of the variation in the infant gut bacterial community structure, respectively. Ellipses represent the confidence intervals at 95%. No significant effects of age or exposure to parasites were detected for the eukaryote community structure. (c and d) Shannon diversity of gut bacterial (c) and eukaryote (d) community structures. A significant difference was detected only for eukaryote community alpha-diversity by Mann-Whitney tests for comparisons between the two groups. (e and f) Estimated richness of gut bacterial (e) and eukaryote (f) community structures. No significant differences were detected by Mann-Whitney tests for Chao1 estimated richness between the parasite-positive and -negative groups.

**(iv) Infants from 1 to 2 years of age.** In older weaned children between 1 and 2 years of age, we detected an effect of parasite presence on the bacterial community structure (Fig. 4). Parasite exposure explained 8.7% of the bacterial variation, whereas age explained 7.7% (Fig. 4a). Chao1 richness and Shannon diversity indices showed no changes in richness or evenness (Fig. 4c and e). Although there was no significant effect detected on eukaryotic beta-diversity (Fig. 4b) and eukaryotic richness (Fig. 4f), eukaryote alpha-diversity (Shannon index) showed a statistically significant increase in the group colonized by parasites (Fig. 4d).

**FIG 5:**
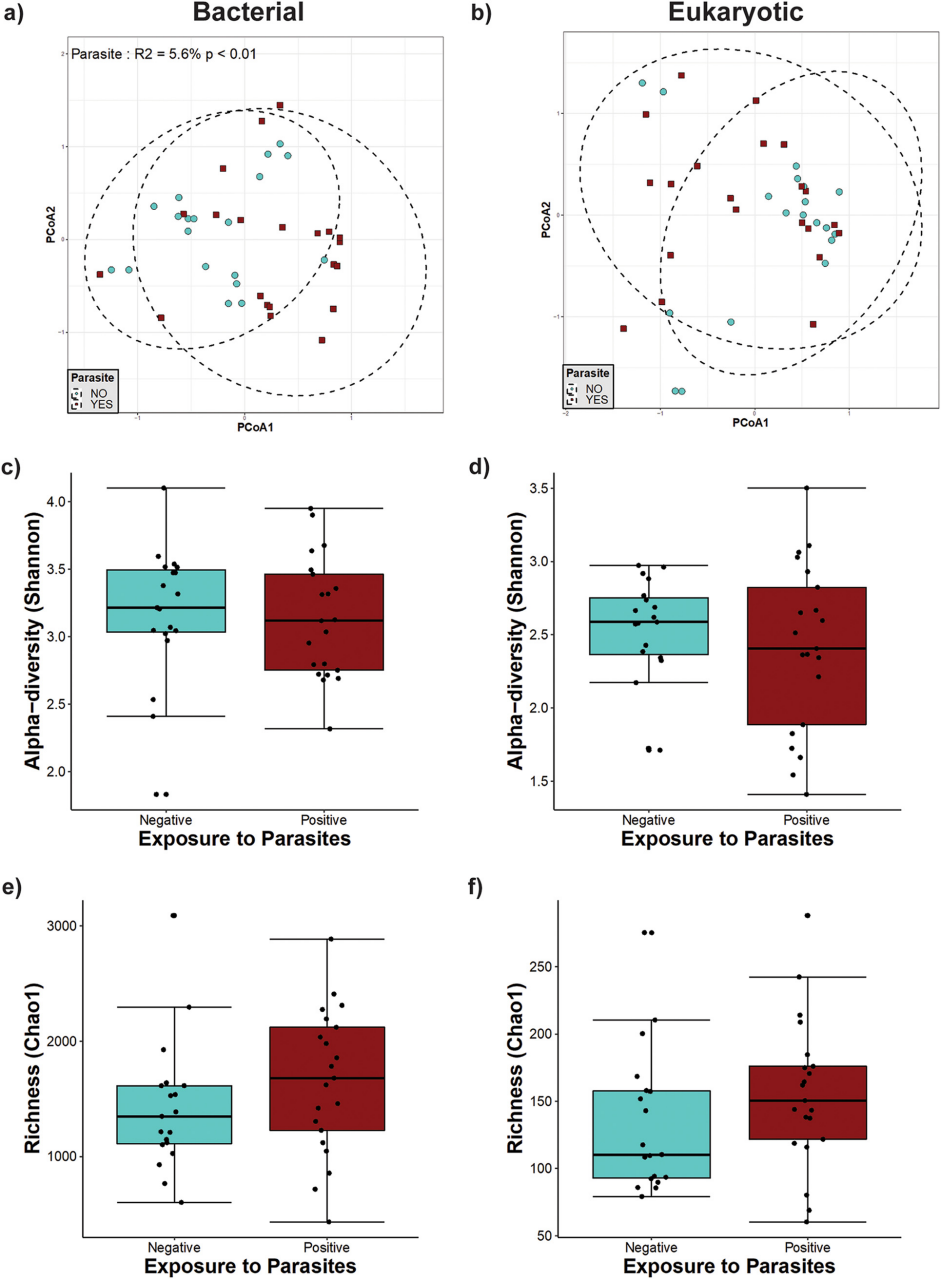
Microbial diversity in mothers. (a and b) Principal-component analysis (PCoA) ordination of the variation in beta-diversity of human gut bacterial (a) and eukaryote (b) communities based on Bray-Curtis dissimilarities. Color and shape represent maternal exposure to parasites (turquoise circles for negative and dark-red squares for positive exposure). PERMANOVAs indicate that exposure to parasites explains 5.6% (*P* < 0.01) of the variation in mother gut bacterial community structure. Ellipses represent the confidence intervals at 95%. No significant effects of age or exposure to parasites were detected for the eukaryote community structure. (c and d) Shannon diversity of gut bacterial (c) and eukaryote (d) community structures. (e and f) Estimated richness of gut bacterial (e) and eukaryote (f) community structures. No significant differences were detected by Mann-Whitney tests for richness and alpha-diversity between the parasite-positive and -negative groups.

In mothers, we also found a significant effect of parasite exposure on bacterial beta-diversity (Fig. 5a), explaining 5.6% of the variation of the community structure, and no effect on eukaryotic diversity (Fig. 5b). No statistically significant effects were observed in mothers exposed to parasites in bacterial and eukaryote community richness (Chao1) and alpha-diversity (Shannon) (Fig. 5c to f).

### Relative abundances of gut bacteria and eukaryotes.

**FIG 6:**
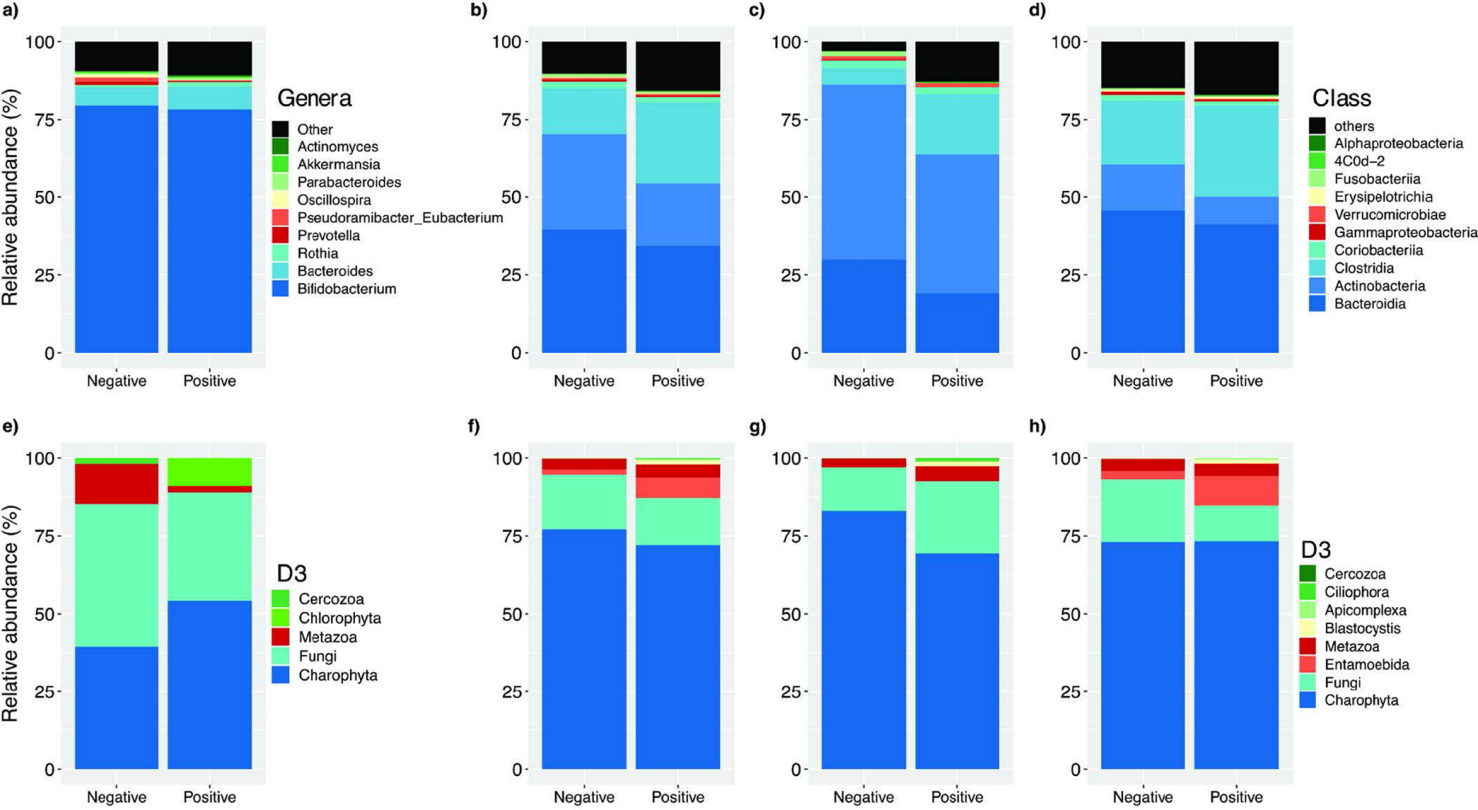
Relative abundances of gut bacterial (a to d) and eukaryote (e to h) compositions of parasite-exposed individuals. (a and e) Bacterial and eukaryote compositions in unweaned infants younger than 5 months of age. (b to d and f to h) Relative abundances depending on parasite colonization in individuals over 1 year of age (b and f), only in weaned infants between 1 and 2 years of age (c and g), and only in mothers (d and h).

Differential abundance analysis revealed changes in bacterial composition associated with parasite colonization and exposure (Fig. 6). In unweaned infants less than 5 months old exposed to parasites, the most abundant genera were *Bifidobacterium* and *Bacteroides* (Fig. 6a), whereas the genera *Pseudoramibacter*, *Eubacterium*, *Prevotella*, and *Oscillospira* were less abundant than in nonexposed infants. We also found decreases in the taxa Metazoa and Fungi in the unweaned infants exposed to parasites (Fig. 6e).

In individuals older than 1 year of age, only weaned children from 1 to 2 years of age, and only the mothers, the most abundant classes were *Bacteroidia*, *Actinobacteria*, *Clostridia*, and *Coriobacteriia*, and the presence of parasites was consistently associated with an increase in the relative abundance of *Clostridia* and decreases in the relative abundances of *Actinobacteria* and *Bacteroidia* (Fig. 6b to d). There were also decreases of *Fusobacteria* and *Gammaproteobacteria* proportions associated with colonization by parasites in the children from 1 to 2 years of age and the mothers, respectively.

Concerning eukaryotes, in individuals older than 1 year of age, there was a decrease of the taxon Fungi (Fig. 6f), whereas in only children from 1 to 2 years of age, there was an increase in Fungi (Fig. 6g), and in only the mothers, Fungi had a lower abundance in parasite-positive individuals (Fig. 6h).

### Eukaryote-bacterium correlations.

**FIG 7:**
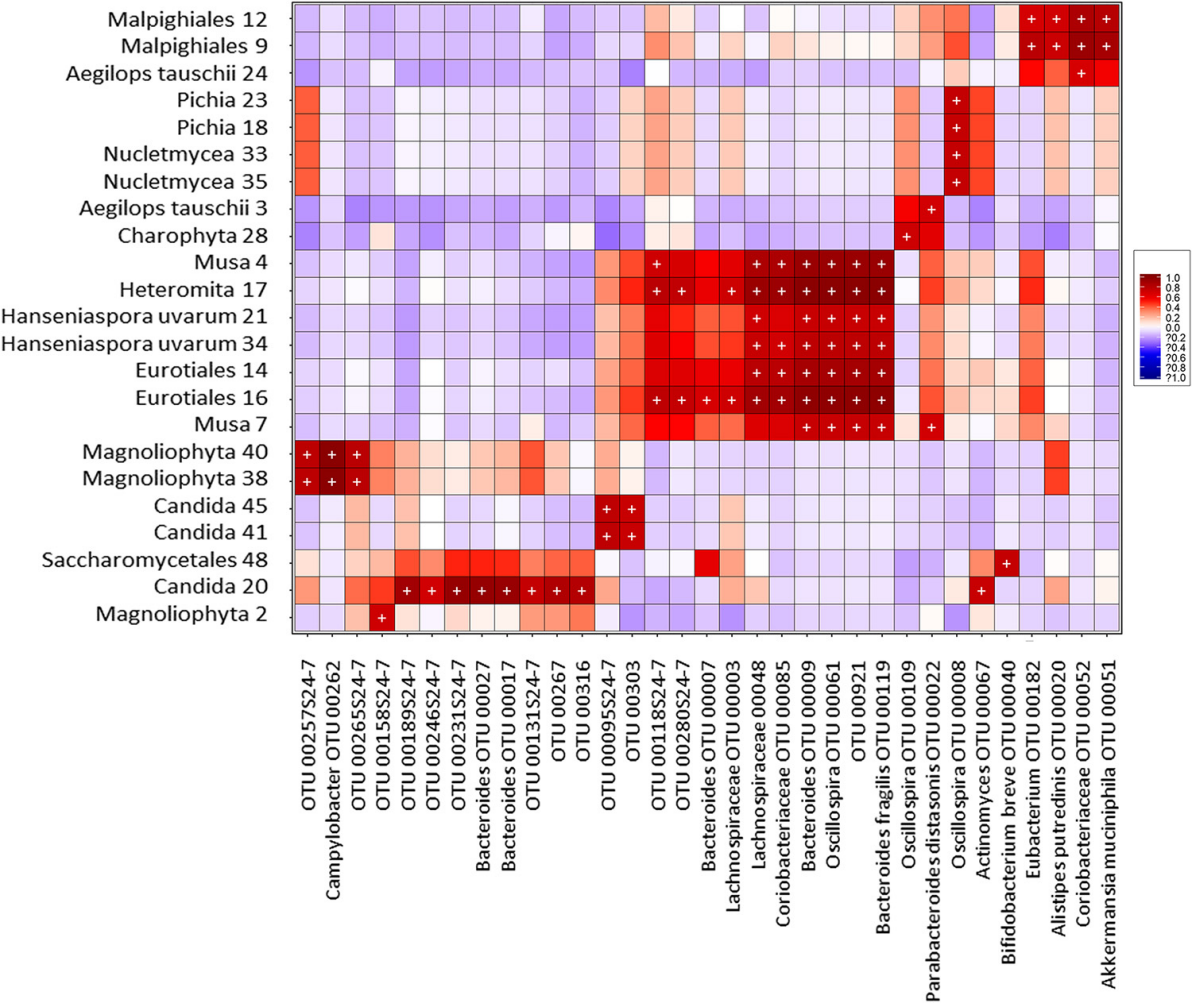
Heat map of biweight correlations (Pearson) between the top 50 bacterial (*x* axis) and the top 50 eukaryote (*y* axis) taxon OTUs in fecal samples of unweaned infants (∼3 to 4 months old). Colors denote positive (red) and negative (blue) correlation values. Significant correlations are denoted with a plus sign (*P < *0.05 [false discovery rate {FDR}]).

**FIG 8:**
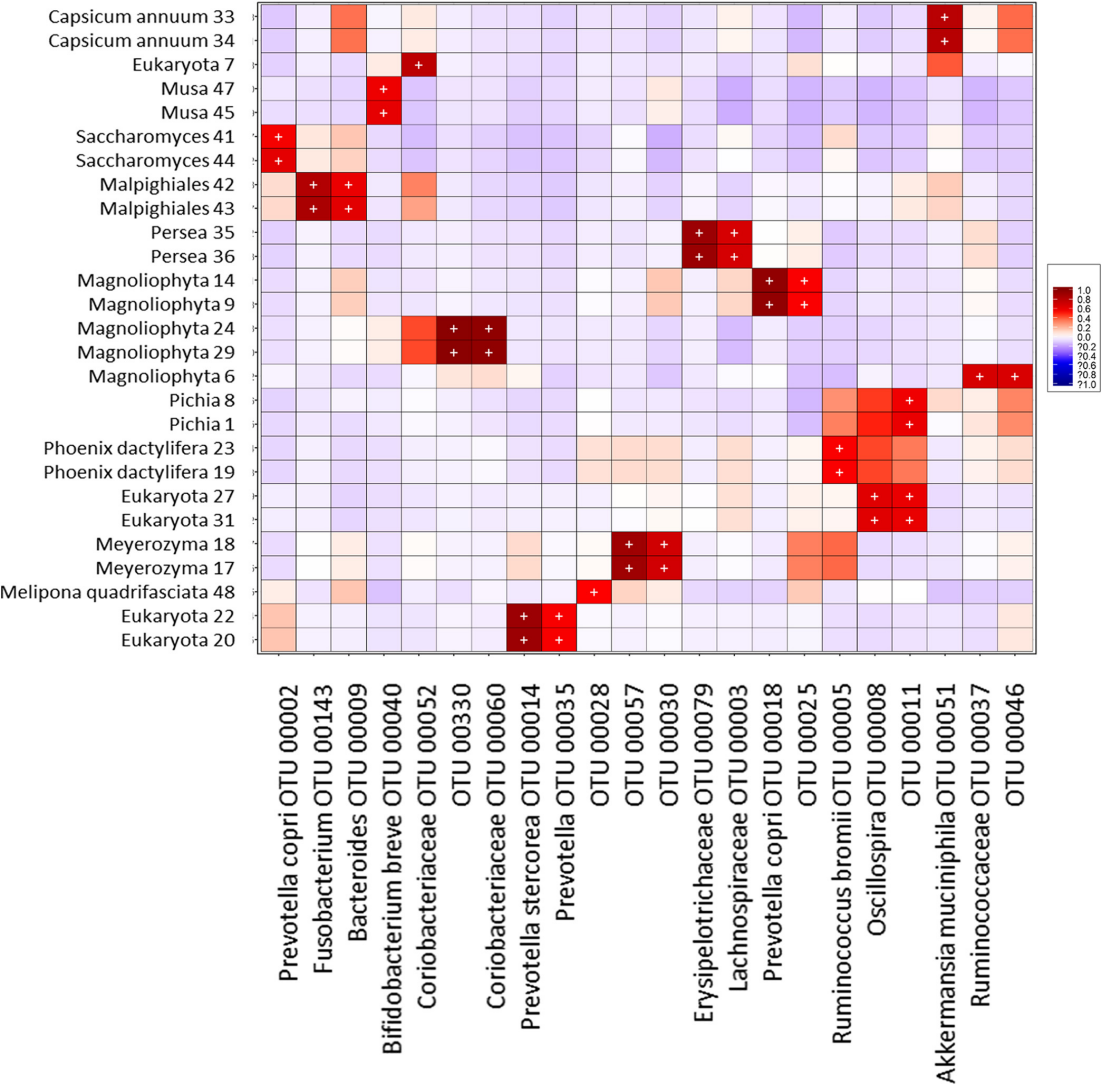
Heat map of biweight correlations (Pearson) between the top 50 bacterial (*x* axis) and the top 50 eukaryote (*y* axis) taxon OTUs in fecal samples of weaned children (>1 year old) and mothers. Colors denote positive (red) and negative (blue) correlation values. Significant correlations are denoted with a plus sign (*P < *0.05 [FDR]).

Given the observed role of parasites in the bacterial community structure, we wanted to know whether additional relationships of other eukaryotes with bacteria were present in the intestine. To look for correlations, we created heat maps of biweight correlations between the top 50 bacterial taxon operational taxonomic units (OTUs) and the top 50 eukaryote OTUs in fecal samples, independently of parasite exposure. In the group of unweaned infants less than 5 months of age, we observed numerous statistically significant correlations between bacteria and eukaryotes (Fig. 7), several of which were from food and environmental origins, whereas in both the group of weaned children older than 1 year of age and the group of mothers, there were fewer correlations (Fig. 8).

We found positive correlations of *Bacteroides*, Bacteroides fragilis, *Lachnospiraceae*, *Coriobacteriaceae*, and *Oscillospira* with the protist *Heteromita*. Remarkably, we found positive correlations of *Oscillospira* with *Entamoeba coli* and Prevotella stercorea with Entamoeba hartmanni. Additionally, we found various positive correlations of bacteria with fungi, such as *Bacteroides* and *Bifidobacterium* with *Candida*, Aspergillus, and *Hanseniaspora*; *Lachnospiraceae* and *Coriobacteriaceae* with Aspergillus and *Hanseniaspora*; *Oscillospira* with *Pichia*, Aspergillus, and *Hanseniaspora*; *Actinomyces* with *Candida*; and Prevotella copri with *Saccharomyces*.

## DISCUSSION

The most recent reports available estimate that 3.5 billion people are colonized by parasites globally (19, 20). Even though eukaryotes are present in much lower abundances than bacteria, it has been demonstrated that monocolonization by parasites is associated with intestinal microbiota composition changes (5, 11, 21–24). Colonization by parasitic eukaryotes usually does not follow a one-host–one-parasite model (25–29), and very few studies have assessed the intestinal microbiota composition when multiple parasites are present (30). In this project, we studied whether exposure to intestinal parasites in an asymptomatic cohort of mother-child binomials from a semirural community in Mexico is related to changes in the bacterial and eukaryotic intestinal microbiota. Our study revealed important changes in bacterial intestinal microbiota relative abundances associated with exposure to parasites, characterized by an increase in the abundance of the taxon *Clostridia* and decreases in the abundances of *Actinobacteria* and *Bacteroidia*, with no important changes in alpha-diversity indices.

The mother’s microbiota has been determined to be an important microbial source during early colonization of the infant gut (13, 31–41); however, we found no binomial identity effect on either bacterial or eukaryote communities. This may be because most of the above-mentioned studies compared infant samples collected shortly after birth, and the samples analyzed in this study were from older infants. However, it is also possible that the increased dissimilarity between mother-infant binomials in this semirural setting may reflect a larger influence of other individuals or other components of the environment rather than vertical transmission, as has been previously reported for the development of microbiome structure and diversity (32, 41, 42).

We found nine different parasites in the binomials studied, predominated by the protists *B. hominis* (20.6%), E. coli (16.3%), *E. nana* (6.5%), and *E. dispar* (5.4%) and the two helminths *A. lumbricoides* (5.4%) and *H. nana* (4.3%). More than half of the exposed individuals (54.8%) were colonized by two or more different parasites. Yet all parasite-positive individuals in our cohort remained asymptomatic. Interestingly, when parasite-positive individuals were compared with parasite-negative ones, we found that the parasite-positive weaned children were significantly older and had a higher proportion of females.

Even though we did not find parasite colonization in the unweaned children, we hypothesize that these infants are exposed to the parasites of their parasite-positive mothers. These exposures originate from birth, breastfeeding, and spending the majority of the time together in the same environment (39). Due to the vertical transmission of bacteria from the mother, the intestinal microbiota of the newborn closely matches the maternal microbiota, and depending on the delivery mode, it resembles the stool, vaginal, or skin microbiota (34–36). It remains unclear why infants in this age group did not become colonized by parasites, but our data clearly indicate that even in the absence of colonization, there are distinct microbiome patterns associated with maternal colonization. These infants were unweaned, and with breastfeeding, the infant bacterial diversity remains limited because the microbiota is dominated by species involved in human milk metabolism in breastfed infants, with greater *Bacteroides* and *Bifidobacterium* abundances (16, 38).

In the first 4 months of gut microbe colonization, the infant microbiota gradually differs from the mother’s microbiota, reflecting the progressively increasing gut microbial diversity and complexity of the growing infant over microbes acquired maternally (31, 35). Although we did not find evidence of parasite colonization in the unweaned children, parasites from their positive mothers might still find their entrance to the infant intestine. The parasites are incapable of colonizing, probably also because of the level of maturation of the intestinal microbiota (36). However, these parasites might be temporarily passing through the infant intestine, which may be long enough to interact with the bacteria of the children’s gut and influence its composition. It is also possible that the bacteria that come into contact with the infant from the mother might have already been selected by the parasites in the mother’s gut. These are just hypotheses at this point and require further research.

Our study detected increases in bacterial and eukaryotic richness and alpha-diversity due to parasite colonization only in unweaned infants over 1 year old. Previous reports have associated colonization by parasites with higher intestinal bacterial diversity. A previous study by Morton et al. (5) in rural populations in Cameroon found that the presence of the protist *Entamoeba* was associated with a significant increase in alpha (intrahost)-diversity. Furthermore, a recent study by our group (11) found dramatic increases in bacterial richness and alpha-diversity in people colonized by *Blastocystis* from the same community as the one of the present study. The discrepancies between this study and the previous ones, which did not include infants, strongly suggest that the effect of parasite exposure on alpha-diversity could be age dependent and may be attributable to the lower alpha-diversity normally found in infant samples (18, 37). This is supported by a recent study in children from Colombia (30), which also found no differences in bacterial alpha-diversity in children positive for parasites. However, we did not observe an increase in alpha-diversity in the parasite-positive mothers. This finding suggests that in addition to age, other factors may play a role. We think that the common multiparasitic colonization found in our settings may lead to more complex interactions with the resident microbiota, which could affect the bacterial alpha-diversity in the intestine. This may be detected only with further studies with a larger population size and by comparing the effects between monoparasitic and multiparasitic colonization.

Despite the discrepancies with other studies in relation to alpha-diversity, we found that parasite exposure was significantly associated with bacterial intestinal microbiota beta-diversity in both infants and mothers in this study but not with the eukaryotic microbiota. In unweaned children younger than 5 months old, parasite exposure explained 14.9% (*P* = 0.003) of the variation in bacterial community structure, whereas in the groups of older weaned infants and mothers, parasite exposure was a weaker driver of beta-diversity. This suggests that while parasite exposure is an important factor shaping intestinal bacterial beta-diversity, its effects are more evident during the earlier stages of gut bacterial community establishment.

Exposure to parasites was also associated with changes in specific taxa, including an increase in the class *Clostridia* and a decrease in the class *Bacteroidia*. This result agrees with the results of a study by Morton et al. (5), in which there was a strong correlation of *Entamoeba* with a higher frequency of *Firmicutes* (particularly the *Clostridia* class) and a lower frequency of *Bacteroidetes* (mostly *Prevotella*) in E. histolytica-positive samples. In the study focused on *Blastocystis* (11), colonization was also associated with significant increases in the genera *Ruminococcus* and *Oscillospira* (members of the *Clostridia* class) and a reduction in *Prevotella* (*Bacteroidia* class).

These changes in relative abundance may be the result of direct parasite-bacterium interactions driven by competition for resources, predation, or the production of molecules that may affect the fitness or survival of the microorganisms involved (1). Protists are well-known bacterivores. *Entamoeba* and *Blastocystis* can graze on bacteria (43, 44), and this is an important mechanism for the top-down control of bacterial communities due to their high feeding rates (45). It was also recently reported that Trichuris muris, a nematode from the mouse gut, acquires its intestinal microbiota from the mouse intestine, very likely through ingestion (46). Furthermore, the mechanisms by which bacteria avoid protist predation change their ability to survive and can even promote the emergence of virulence and invasion (45, 47).

Parasites may also influence the bacterial community structure in the intestine through indirect interactions with bacteria. Some protists like *Entamoeba* and Giardia, which have mucolytic enzymes (48, 49), and helminths like Trichuris trichiura, which stimulate mucin expression or express mucin-like molecules themselves (50–52), can alter the outer mucus layer, changing the bacterial microenvironment and sources of nutrition for certain taxa. Additionally, the parasites may produce metabolites that could influence the regulation of the immune system, a mechanism commonly used in helminths. Trichuris muris and Heligmosomoides polygyrus bakeri can induce the generation of regulatory T cells (Tregs) (53, 54), changing the physical microenvironment by modifying mucus and antimicrobial peptide production, thus potentially promoting the outgrowth of specific taxa among the members of the microbiota (55). The bacterial and eukaryotic taxa identified in this study and other research should be studied in appropriate animal models to further determine the mechanisms involved in these multikingdom interactions.

The effect of exposure to parasites on intestinal eukaryote relative abundance was not as strong as it was with bacteria. However, there were some changes in the abundances of the taxa Chlorophyta, Charophyta, and Fungi, which proved to be age dependent. The correlation analysis identified several significant positive associations between other intestinal eukaryotes and bacteria, most of which were with eukaryotes from food or environmental sources, such as Cucurbita pepo, Capsicum annuum, *Musa*, *Characium* sp., and *Chlorophyceae*. However, among the eukaryotes identified in the sequencing analysis that are known to be gut residents (*Entamoeba coli*, *E hartmanni*, *Iodamoeba*, *E. dispar*, *Ascaris*, Trichuris trichiura, *Hymenolepis nana*, and *Blastocystis*), we observed positive correlations of two species with bacteria, *Entamoeba coli* with *Oscillospira* and *E. hartmanni* with Prevotella stercorea.

Remarkably, the strongest positive correlations found in the present study were between bacteria and several common fungi of the intestinal microbiota, even though fungal and bacterial abundances in the gut have been negatively correlated, and disruption of the bacterial microbiota is a condition required for fungal overgrowth (56). Agonistic and antagonistic relationships have been described between intestinal fungi and bacteria (56–61). It has been shown that the common yeast Candida albicans suppressed the regrowth of *Lactobacillus* and promoted the recovery of *Bacteroidetes* populations during antibiotic recovery (62). Our study also detected the co-occurrence of *Candida* species with *Bacteroides*. Other previously unreported co-occurrences of bacteria and fungi were found, such as *Oscillospira* with Aspergillus, *Pichia*, and *Hanseniaspora*; *Actinomyces* and *Bifidobacterium* with *Candida*; and Prevotella copri with *Saccharomyces.* The mechanisms and functions of these interactions in the gut deserve further studies.

Our work supports previous reports that the presence of intestinal parasites is linked to strong bacterial microbiota community changes. By including mother-child binomials, our work further revealed that these effects occur even in the absence of direct colonization in infants, strongly suggesting that the effect of parasite colonization on the microbiome may also lead to changes in the vertical transmission of bacterial taxa. This implies that colonization by parasites may be an important indirect factor in the inheritable features of the human gut microbiome. How these intestinal microbiota changes associated with parasites may modify the immune system and other aspects of metabolism remains to be elucidated.

## MATERIALS AND METHODS

### Study population, study design, and ethical considerations.

Xoxocotla is a semirural community of the State of Morelos, Mexico, located 120 km south of Mexico City (longitude, 99°19W; latitude, 18°3N) in an area spanning 29,917 km^2^ with a tropical climate (warm subhumid). The total population is 5,163 people, whose main sources of income are agriculture and commerce. Sample collection was carried out between April 2011 and January 2013.

In this cross-sectional study of cohorts, every volunteer mother was informed about the characteristics of the project, the objectives, and the advantages of participation, along with the biological samples needed, the sampling procedures, and possible complications that could arise. All the participant mothers signed a written informed consent letter for their children and themselves prior to sample collection. Afterwards, questionnaires to collect sociodemographic, socioeconomic, and health datum antecedents (Rome III questionnaire for gastrointestinal symptoms [63]); nutrition data; and way of delivery and to ensure that there was no use of antibiotics and other drugs for at least 6 months prior to sampling were applied by nurses at the community of Xoxocotla, Morelos, Mexico. All variables were recorded in a database.

All procedures in this study fulfilled the Reglamento de la Ley General de Salud en Materia de Investigación para la Salud of Mexico, in particular the chapters about the ethical aspects of research in human beings, research in communities, research in minors, and research in women of fertile age and pregnant women (Diario Oficial de la Federación, Febrero 1984). All methods were approved by the Ethics Committee of the Faculty of Medicine of the National Autonomous University of Mexico, and research was carried out in accordance with the Declaration of Helsinki.

### Sample collection, parasite detection, and DNA extraction from fecal samples.

Fecal samples from mother-child binomials that fulfilled the criteria were collected in sterile plastic containers, immediately placed at 4°C for transport to the laboratory, and stored at −20°C until analysis. The fecal samples were submitted to stool microscopic analysis for the detection of intestinal parasites for the construction of the parasitized and nonparasitized cohorts. The presence of the main intestinal parasites historically found in Xoxocotla (E. histolytica/*E. dispar*, E. coli, *B. hominis*, *I. butshlii*, *E. nana*, C. mesnili, Giardia intestinalis, Cryptosporidium parvum, *A. lumbricoides*, and *H. nana*) was tested in fecal samples by microscopy.

DNA from fecal samples was extracted as previously described (11). Briefly, samples with 50 mg of stool were mechanically lysed using Mo Bio dry bead tubes (Mo Bio Laboratories, Inc.) in a FastPrep homogenizer (MP Biochemicals). DNA isolation was performed using the QIAamp Fast DNA stool minikit (Qiagen) according to the manufacturer’s instructions.

The presence of E. histolytica/*E. dispar*, *B. hominis*, G. intestinalis, and C. parvum was confirmed by quantitative PCR (qPCR) as previously reported (11). Briefly, qPCR was performed on an Applied Biosystems 7500 machine using QuantiTect SYBR green master mix (Qiagen) in 10-μl reaction mixture volumes with 6.25 pmol each of primers Ehd-239F–Ehd-88R, BhRDr-RD5, Giardia-80F–Giardia-127R, and CrF-CrR (see Table S1 in the supplemental material). The amplification conditions consisted of 35 cycles of 1 min each at 94°C, 59°C, and 72°C, with an additional step of 95°C for 15 s, 60°C for 1 min, 95°C for 30 s, and 60°C for 15 s (64). Samples previously known to be positive for each parasite as well as standard curves using DNA from each parasite from the ATCC’s enteric protist DNA panel were included as positive controls in the qPCR plates. The difference between the average cycle threshold (*C_T_*) value of each parasite qPCR and the average *C_T_* value of the 18S rRNA gene reaction was calculated to determine the parasitic loads in each sample.

10.1128/mSphere.00083-21.2 TABLE S1 Primer sequences used for protist determination by qPCR. Download Table S1, DOCX file, 0.03 MB. Copyright © 2021 Partida-Rodriguez et al. 2021 Partida-Rodriguez et al. https://creativecommons.org/licenses/by/4.0/ This content is distributed under the terms of the Creative Commons Attribution 4.0 International license.

### Determination of fecal bacterial composition.

The DNA from 46 mother-child binomial fecal samples, isolated as described above, was used for the sequencing of microbial communities. For bacterial determination, samples were amplified by PCR in triplicate using barcoded primer pairs flanking the V4 region of the 16S rRNA gene as previously described (65, 66). Each 50-μl PCR mixture contained 22 μl of water, 25 μl of TopTaq master mix (Qiagen), 0.5 μl of each forward and reverse barcoded primer (66), and 2 μl of template DNA. To ensure that no contamination occurred, controls without template DNA were included. Amplification was performed with an initial DNA denaturation step at 95°C (5 min), 25 cycles of DNA denaturation at 95°C (1 min), an annealing step at 50°C (1 min), an elongation step at 72°C (1 min), and a final elongation step at 72°C (7 min). Amplicons displaying bands at ∼250 bp on a 2% agarose gel were purified using the QIAquick PCR purification kit (Qiagen). Purified samples were quantified with PicoGreen (Invitrogen) in a Tecan M200 plate reader (excitation at 480 nm and emission at 520 nm).

For 16S rRNA gene sequencing, each PCR pool was analyzed on the Agilent Bioanalyzer using the high-sensitivity double-stranded DNA (dsDNA) assay to determine the approximate library fragment size and verify library integrity. Pooled-library concentrations were determined using the TruSeq DNA sample preparation kit, version 2 (Illumina). Library pools were diluted to 4 nM and denatured into single strands using fresh 0.2 N NaOH. The final library loading concentration was 8 pM, with an additional PhiX spike-in of 20%. Sequencing was carried out using a HiSeq 2000 bidirectional Illumina sequencing and cluster kit, version 4 (Macrogen, Inc.).

### Determination of fecal eukaryotic composition.

The composition of eukaryotic microorganisms was determined by 18S rRNA gene sequencing. DNA samples were sent to the Integrated Microbiome Resource at Dalhousie University for amplification and sequencing. The 18S rRNA gene was amplified with the primers E572F (5′-YGCGGTAATTCCAGCTC-3′) and E1009R (5′-AYGGTATCTRATCRTCTTYG-3′), and the reaction mixture included a peptide-nucleic acid blocking primer (5′-TCTTAATCATGGCCTCAGTT-3′) to reduce the amplification of mammalian sequences. Amplification was carried out in duplicate, with one reaction mixture using undiluted DNA and the other using DNA diluted 1:10 in PCR water. Amplification was conducted according to previously described protocols (67). PCR products were visualized on E-gels, quantified using Invitrogen Qubit with PicoGreen, and pooled at equal concentrations, according to a previous report (68). PhiX was spiked in at 5%, and the resulting library was sequenced at Dalhousie University on the Illumina MiSeq instrument using the MiSeq 500-cycle reagent kit, version 2 (250 by 2).

### Bioinformatics analysis.

Sequences were preprocessed, demultiplexed, denoised, quality filtered, and trimmed and chimeras were removed using Mothur for the 16S rRNA gene (68) or QIIME for the 18S rRNA gene (69).

### (i) 16S.

All sequences were processed using Mothur according to standard protocols as previously described (68). Quality sequences were obtained by removing sequences with ambiguous bases, a quality read length, and/or chimeras identified using chimera.uchime. Quality sequences were aligned to the SILVA bacterial reference alignment, and OTUs were generated using a dissimilarity cutoff of 0.03. Sequences were classified using the classify.seqs command.

### (ii) 18S.

Demultiplexed reads were trimmed to a uniform length of 250 bp using the FastX-Toolkit (http://hannonlab.cshl.edu/fastx_toolkit/) and clustered into OTUs using the minimum entropy decomposition (MED) method (70) as implemented in the oligotyping microbial analysis software package (71). MED performs *de novo* taxonomic clustering using Shannon entropy to separate biologically meaningful patterns of nucleotide diversity from sequencing noise; the processed data are partitioned into phylogenetically homogeneous units (MED nodes) for downstream microbial eukaryotic diversity analyses. This analysis was carried out with the minimum substantive abundance parameter (-M) set at 250 reads. All other parameters were run with default settings; the maximum variation allowed per node (-V) was automatically set at 3 nucleotides.

Representative sequences were classified by clustering against the Greengenes database at 97% similarity (16S rRNA gene [72]) or SILVA release 123 at 99% similarity (18S rRNA gene [73]). The 16S rRNA gene data set was filtered to remove mitochondrion and chloroplast sequences and OTUs present in fewer than three samples. The 18S rRNA gene data set was filtered to remove mammalian and plant sequences and all OTUs present in fewer than three samples. The final data sets contained 5,458,311 and 2,443,357 quality sequences for 16S and 18S, respectively. The 16S data set showed a mean of 64,215 (range, 2,119 to 228,403) sequences per sample identified as 3,046 bacterial OTUs. Samples contained a mean of 508 (range, 180 to 1,073) bacterial OTUs per sample. The 18S data set showed a mean of 28,085 (range, 16 to 223,045) sequences per sample identified as 694 eukaryote OTUs. Samples contained a mean of 86 (range, 6 to 244) eukaryote OTUs per sample.

### Statistical analysis.

Differences in frequencies for categorical and continuous variables between cases and controls were evaluated using chi-squared and Student’s *t* tests, respectively.

Microbial alpha- and beta-diversities as well as the relative abundances of bacterial and eukaryotic taxa were computed using phyloseq (74), along with additional R-based computational tools (75–81). Principal-component analyses (PCoAs) were employed to visualize variation in the microbial community structure (previously transformed by variance-stabilizing transformation to ensure that our statistical results were not an artifact of heteroscedastic dispersion between groups [74]). We quantified the relative influence of various drivers of intestinal microbial beta-diversity by conducting permutational multivariate analysis of variance (PERMANOVA) on Bray-Curtis dissimilarities. The Shannon alpha-diversity and Chao1 richness indices were calculated using phyloseq without excluding singletons and doubletons, and differences between groups were statistically tested by a Mann-Whitney test.

The R packages DESeq2 (81) and MaAsLin (82) were used to calculate differentially abundant OTUs. Correlation analysis was performed using the bicor method in the R package microbiome to correlate the 100 most abundant OTUs from the 16S and 18S rRNA gene data sets. Features in the analysis were included as OTUs and as OTUs combined into taxonomic families.
