# Supplementary figures and images for: Exposure to Parasitic Protists and Helminths Changes the Intestinal Community Structure of Bacterial Communities in a Cohort of Mother-Child Binomials from a Semirural Setting in Mexico
Source: mSphere. 2021 Aug 18;6(4):e00083-21. doi: 10.1128/mSphere.00083-21 (PMC8386383; doi:10.1128/mSphere.00083-21)

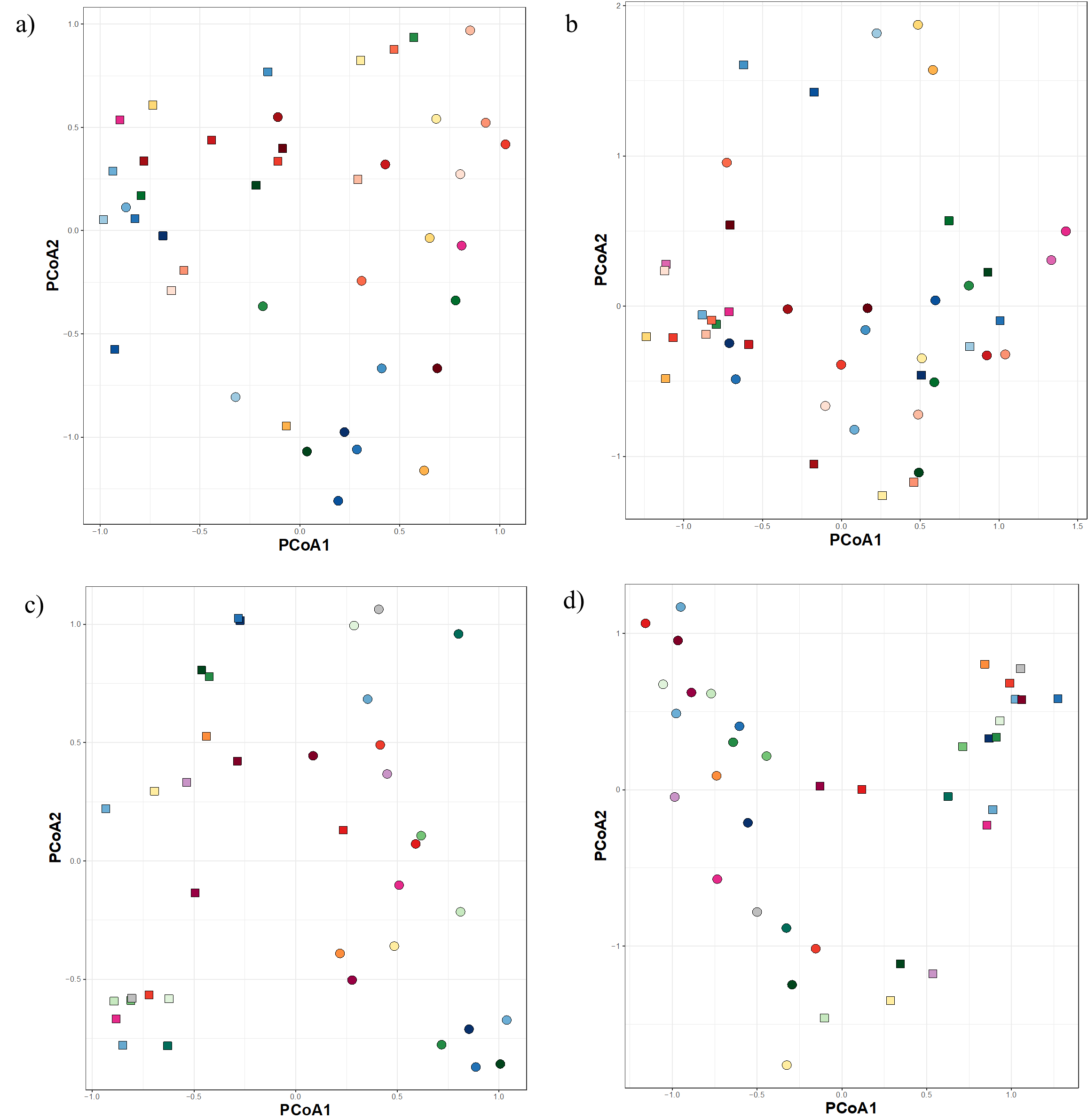

Supplement: FIG S1 [file msphere.00083-21-sf001.tif]
