# Supplementary material for: Exposure to Parasitic Protists and Helminths Changes the Intestinal Community Structure of Bacterial Communities in a Cohort of Mother-Child Binomials from a Semirural Setting in Mexico
Source: mSphere. 2021 Aug 18;6(4):e00083-21. doi: 10.1128/mSphere.00083-21 (PMC8386383; doi:10.1128/mSphere.00083-21)
Supplement: TABLE S1 [file msphere.00083-21-st001.docx]

| Primer | Sequence | Reference |
| --- | --- | --- |
| *B. hominis*  BhRDr  RD5 | 5’ GAG CTT TTT AAC TGC AAC AAC G 3’  5’ ATC TGG TTG ATC CTG CCA GT 3’ | 1  2 |
| *E. histolytica/dispar*  Ehd-239F  Ehd-88R | 5’ ATT GTC GTG GCA TCC TAA CTC A 3’  5’ GCG GAC GGC TCA TTA TAA CA 3’ | 3 |
| *G. duodenalis*  Giardia-80F  Giardia-127R | 5’ GAC GGC TCA GGA CAA CGG TT 3’  5’ TTG CCA GCG GTG TCC G 3’ | 3 |
| *C. parvum*  CrF  CrR | 5’ CGC TTC TCT AGC CTT TCA TGA 3’  5’ CTT CAC GTG TGT TTG CCA AT 3’ | 3 |
| 18S rRNA  F  R | 5’ GTA CAC ACC GCC CGT C 3’  5’ TGA TCC TTC TGC AGG TTC ACC TAC 3’ | 4 |

1. Scicluna SM, Tawari B, Clark CG. Protist. 2006 Feb;157(1):77-85.

2. Clark CG. Mol Biochem Parasitol. 1997 Jul;87(1):79-83.

3. Verweij JJ, Blangé RA, Templeton K, Schinkel J, Brienen EA, van Rooyen MA, van Lieshout L, Polderman AM. J Clin Microbiol. 2004 Mar;42(3):1220-3.

4. Nieves-Ramírez ME, Partida-Rodríguez O, Laforest-Lapointe I, Reynolds LA, Brown EM, Valdez-Salazar A, Morán-Silva P, Rojas-Velázquez L, Morien E, Parfrey LW, Jin M, Walter J, Torres J, Arrieta MC, Ximénez-García C, Finlay BB. mSystems. 2018 Jun 26;3(3).
